# Primary Care Utilization and Prehospital Emergency Demand Among Patients with Multimorbidity in Spain

**DOI:** 10.3390/nursrep15110377

**Published:** 2025-10-24

**Authors:** Enrique Coca-Boronat, José Miguel Morales-Asencio, Daniel Coca-Gallen, Laura Gutiérrez-Rodríguez, Inmaculada Lupiáñez-Pérez, Cristina Guerra-Marmolejo, José Sáenz-Gómez, Bibiana Pérez-Ardanaz

**Affiliations:** 1 Health Care Emergency Center 061, 29590 Málaga, Spain; enrique.coca.sspa@juntadeandalucia.es; 2 Department of Nursing, Faculty of Health Sciences, University of Málaga, 29071 Málaga, Spain; jmmasen@uma.es (J.M.M.-A.); inmalupianez@uma.es (I.L.-P.); cguerra@uma.es (C.G.-M.); 3 Instituto de Investigación Biomédica de Málaga y Plataforma en Nanomedicina (IBIMA Plataforma BIONAND), 29590 Málaga, Spain; bibianap@ugr.es; 4 Department of Critical Care, Hospital Universitario de Valladolid, 47003 Valladolid, Spain; 5 Primary Health Care District of Málaga, 29009 Málaga, Spain; 6 Health Care Emergency Center 061, 41092 Sevilla, Spain; 7 Faculty of Health Sciences, University of Granada, 51001 Ceuta, Spain

**Keywords:** emergency medical services, multimorbidity, primary health care, utilization review, continuity of patient care

## Abstract

**Background/Objectives:** Patients with multimorbidity frequently rely on emergency services when continuity of care is weak. Strengthening communication between emergency and primary care can prevent unnecessary hospitalizations, yet this relationship remains underexplored. The aim of this study was to analyze the relationship between primary health care utilization in patients with multimorbidity and their demand for prehospital emergency services. **Methods:** An observational, longitudinal, analytical, and retrospective study was conducted in Málaga (Spain) between 2013 and 2017. Adults (>18 years) with multimorbidity who requested prehospital emergency care services at home were included; those with cancer, rare diseases, severe mental disorders, or incomplete electronic records were excluded. Variables encompassed sociodemographic, clinical, and behavioral characteristics, comorbidities, functional status, polypharmacy, resource type, and outcomes (on-site resolution or hospital referral). Primary health care visits before and after prehospital emergency use were extracted from electronic records. Descriptive, bivariate, and multivariate analyses were performed. **Results:** Among 532 patients, prior primary health care attendance predicted subsequent utilization (β = 0.57; *p* < 0.001), along with caregiver availability (β = 0.12; *p* = 0.001) and prehospital emergency services hyper-demand (β = 0.08; *p* = 0.022). Super-utilizers were younger, had ≥4 comorbidities, polypharmacy, prior family medicine visits, home oxygen therapy, and lower substance or alcohol use. **Conclusions:** In multimorbid adults, prehospital emergencies demand is influenced by factors beyond severity, including comorbidities, polypharmacy, the use of home medical devices, caregiver availability, and primary health care utilization patterns. Strengthening coordination between prehospital emergencies and primary health care, promoting patient–caregiver education, and implementing early notification pathways may improve care continuity and reduce avoidable emergencies.

## 1. Introduction

Management, support, and guidance from primary health care (PHC) are essential for people with multimorbidity. Due to its longitudinal nature, PHC is the best setting to promote patients and caregivers’ participation in complex decision-making, and lifestyle modification [1]. There is a broad consensus on the need for coordination and integration between levels of care, particularly hospital, outpatient, and primary care, to ensure continuity for patients with multimorbidity. However, current models often fail to provide optimal responses [2,3,4,5]. Fragmented care, excessive resource use, and polypharmacy are common consequences [4,6,7]. When communication and collaboration between professionals and patients break down [8], many patients end up using acute services, especially hospital emergency departments (HEDs) [9]. In many cases, this begins with the activation of prehospital emergency care services (PECS), which have had to gradually adapt to these new scenarios [10]. In Spain, PECS are publicly provided and include the “emergency team” model (ET), comprising a physician, an emergency nurse, and an emergency technician. In Andalusia (Southern Spain), “Advanced Coordination Teams” (ACT) operate as an alternative model led by an advanced practice nurse and an emergency technician. Care for one modality or another is decided by the Emergency Coordination Centre based on the type of demand and available resources [11].

Feedback between HEDs, PECS, and PHC, strengthens continuity of care and may prevent repeated visits to HEDs and PECS [2,12]. PHC (including after-hours primary care) and HEDs utilization have been widely studied [13,14,15]. Interventions aimed at improving PHC accessibility or transitional care after HED discharge have yielded inconclusive results in HED readmissions after ED discharge [16]; moreover, the evidence on the association between PHC accessibility or PHC-centered interventions and HED utilization remains inconsistent [17]. Substantial variation exists in HED visits per 1000 population across six countries in Europe and Australia (range from 156 to 311), despite different strategies to expand PHC accessibility to urgent care services [18].

In contrast, the relation between PECS and PHC in multimorbid patients is poorly understood. Prehospital teams increasingly respond to non-urgent, complex chronic cases, often compensating for PHC saturation and HED overcrowding [19,20]. Yet evidence on how these services interact in chronic care is scarce, and no studies in Spain have analyzed utilization patterns or related factors linking PECS and PHC in this context. In Andalusia, 15% of the population using the public health system are classified as high complexity and 5% as very high complexity. These individuals have greater care needs, higher service use, and more comorbidities. They account for 80.2% of PHC visits, 69.7% of PHC emergencies, and 73.4% of HED visits, although specific data on multimorbid patients using PECS in Andalusia are not yet available [21].

Lack of coordinated follow-up for chronically ill patients results in repeated, often avoidable, calls to emergency services, worsened by poor medication adherence, polypharmacy, lack of adequate caregiving support, and functional decline—especially in patients over 65 years of age, who represent a large proportion of PECS users [22,23]. This gap highlights the need to explore PECS use among patients with chronic conditions and the factors influencing it.

Andersen’s conceptual model of health services utilization [24], applied in chronic care, mental health, primary care, or palliative settings, has also been adapted for older people [25,26]. It encompasses three domains: predisposing factors (slightly modifiable by health agents, such as sociodemographic characteristics and health beliefs), facilitating factors (modifiable by health agents, such as family support, community environment, and accessibility to health services), and health condition factors (perceived health, comorbidity, or need for support devices). This study analyzes the relationship between PHC attendance in the population and chronic processes prior to the use of PECS and to verify whether the pattern of use of PHC services changes in the six months after receiving PECS.

## 2. Materials and Methods

### 2.1. Design

An observational, longitudinal, analytical, and retrospective study was carried out during the period 2013–2017, on the assistance provided by the PECS in the city of Málaga, belonging to the Andalusian Health Service (Spain), with 570,000 inhabitants. The study was reported in accordance with the STROBE checklist.

### 2.2. Study Population

Subjects were over 18 years of age, with multimorbidity, who requested PECS to be provided at their home (hospice and home care settings were not included). Patients with underlying cancer, patients with rare diseases or severe mental disorders (psychosis of any etiology and schizophrenia), and those who did not have an attendance report in electronic format were excluded.

Considering the population over 18 years of age and the use of PHC services in Málaga in 2016 (6.77 total attendances per inhabitant), to detect a difference of four visits in the total frequency of PHC attendances in the six months between hyper-users of PECS (those patients who use PECS more than three times in a year) and the rest, with a standard deviation (SD) of 15, a power of 80% and an alpha of 0.05, 506 subjects were needed, which was increased by 5% to cover possible sample losses. The sample was randomly drawn from the total of 75,491 attendances performed by EMS resources in the study period.

### 2.3. Outcomes and Data Collection

Type of PECS resource used (ET or ACT), polypharmacy (defined as the use of ≥5 medications) [27], and functional capacity (assessed using the Barthel Index [28]) were considered. Additional variables included type of clinical process and knowledge of and adherence to the therapeutic regimen, assessed using the Nursing Outcomes Classification (NOC) indicators: 1813 *Knowledge: Treatment Regimen* and 1623 *Compliance Behavior: Prescribed Medication* [29]. The presence of medical technical devices was also checked. Risk behaviors or conditions (smoking, obesity, alcohol and/or substance use, loneliness), hyper-utilization of PECS (defined as three or more visits during the study period), and resolution of care (on-site or referral to a Hospital Emergency Department [HED]) were also recorded. Socioeconomic variables included age, gender, presence of a family caregiver, and living alone. No formal definition of PECS hyperutilization is available. Previous studies have inconsistently defined frequent HED use, most often as four or more visits per year. Since non-multimorbid patients may be more likely to use HEDs, this threshold was lowered to three visits, as a utilization cutoff for a service less frequently used by non-multimorbid patients [30].

Multimorbidity was defined according to the criteria established by the Andalusian Health Service, which identifies adult patients with complex health needs due to chronic conditions associated with organ or system dysfunction and health or social complexity [31]. Visits to PHC teams were recorded based on the type of provider: family physician (FP), primary health care nurse (PHCN), case manager nurse (CMN), and social worker (SW), during the six months before and after the delivery of PECS

The main data source was the mobile electronic clinical record (MECR) used by the PECS. The data on PHC attendance were provided by the Information and Communication Technology Service Management Centre (ICTSMC) of the Andalusian Health Care Service. The research team contacted the ICTSMC to obtain authorization for collating the sample data. A global database with retrospective variables included in the study protocol was provided by ICTSMC; following, the research team analyzed the integrity and consistence of data, debugging issues and guaranteeing the anonymization of data.

### 2.4. Statistical Analysis

All analyses were performed using SPSS 25 and Jamovi 1.6.9. Descriptive statistics were applied to evaluate the overall distribution of the main outcomes. Means and standard deviations or medians and interquartile ranges were used for continuous variables, and percentages for categorical variables. Statistical significance was set at *p* < 0.05.

Subsequently, bivariate analyses were conducted using the chi-square test for categorical variables, and Mann–Whitney U and Wilcoxon signed-rank tests for comparisons of continuous data, as appropriate. Normality of distributions was evaluated with Kolmogorov–Smirnov test.

Multivariable analyses were conducted, with predictor selection based on variables showing significant associations in the bivariate analyses, as well as on the theoretical components of Andersen’s behavioral model.

Multivariable logistic regression models were developed to examine the association between patient-related factors and the utilization of primary health care (PHC) services in the previous six months among individuals using PECS. Adjusted odds ratios (aORs) and their corresponding 95% confidence intervals (CIs) were calculated for each predictor. Model fit and assumptions for logistic regression were evaluated using the Hosmer–Lemeshow goodness-of-fit test, Nagelkerke’s R^2^, and the area under the ROC curve (AUC) to assess model discrimination. Additionally, multicollinearity was assessed using the variance inflation factor (VIF) and tolerance indices.

In the case of the multivariable linear regression models, constructed to assess factors associated with the use of PHC services after receiving care from PECS, the following assumptions were examined: linearity, assessed through partial regression plots; independence of residuals, verified via the Durbin–Watson statistic; homoscedasticity, examined using a scatterplot of standardized predicted values against standardized residuals; and normality of residuals, evaluated using both a histogram and a normal probability plot (P–P plot). Multicollinearity was also assessed by calculating tolerance values, VIF, and by applying mean-centering of variables, where appropriate.

### 2.5. Ethical Approval

The study was approved by the Málaga Provincial Research Ethics Committee (0587-N-17) and authorized by the PECS Board Director.

## 3. Results

The sample consisted of 532 subjects. The general characteristics of the population analyzed (Table 1) provide a median age of 73 years (IQR 18) and a distribution of 52.3% women and 47.7% men. A total of 365 patients (68.6%) required transfer to the hospital, with 61.5% (n = 327) being treated by ETs, and 38.5% (n = 205) by ACTs.

### 3.1. Health Services Utilization

**Figure 1:**
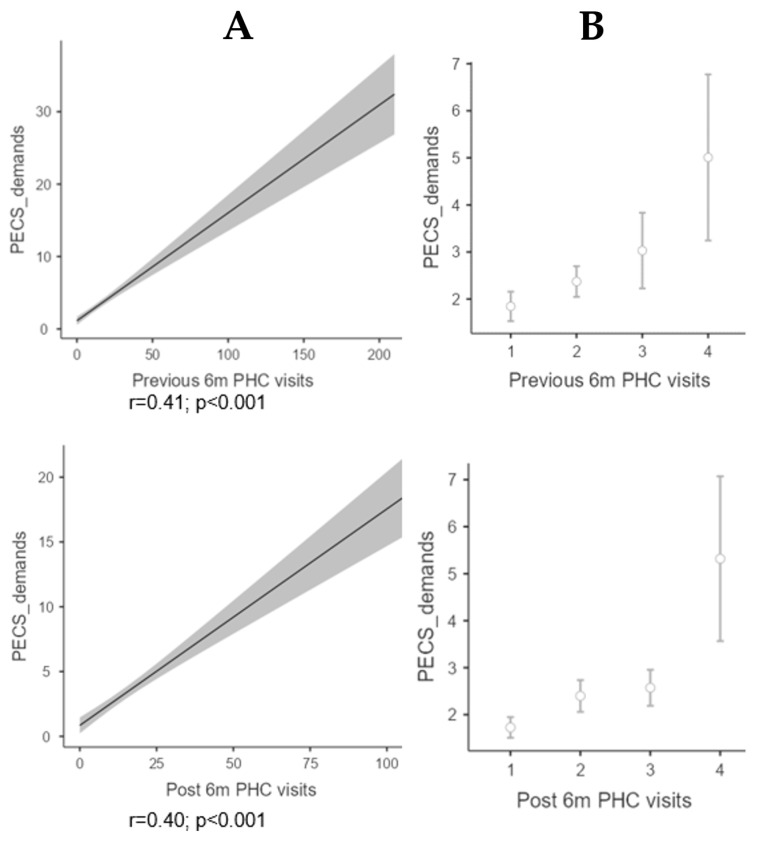
PHC frequentation before and after PECS utilization: (**A**) Correlation between PECS demand and PHC visits; (**B**) Frequency of PHC visits (95% CI).

Regarding the number of visits to the PHC, there were no significant differences in the median number of total visits before (9; IQR: 12) and after (10; IQR: 13.7) having used the PECS (Cohen’s d: −0.02 95% CI: −0.109 to 0.06; *p* = 0.579). The analysis of the attendance at PHC in the six months before and after showed a significant correlation with the demand for PECS (Figure 1). The analysis of paired data to see possible differences between PHC visits before and after requesting PECS did not show that they were significant: z = 55266; *p* = 0.119 (Cohen’s d: −0.02 95% CI: −0.11 to 0.06).

The most frequented PHC providers were GPs and PHCNs, both in the months before and after receiving care from PECS. The median of the total number of visits made to PHC services by hyper-demanders during the six months before and after it was significantly higher compared to non-hyper-demanders, with a low–moderate effect size (Table 2).

Significant differences were observed regarding the type of resource used (ET vs. ACT). Hyper-demanding patients seen by ETs were nearly twice as likely to request PECS (OR = 1.93; 95% CI: 1.18–3.15; χ^2^ = 6.90; *p* = 0.009). In contrast, patients receiving ACT services were less likely to be classified as PECS hyper-demanders (OR = 0.45; 95% CI: 0.22–0.95; χ^2^ = 4.52; *p* = 0.033). To explore possible explanations for these differences, clinical profiles were analyzed. Patients attended by ACT were ten times more likely to present with cardiovascular rather than respiratory conditions (OR = 10.2; 95% CI: 4.04–25.94). However, this association did not remain significant in the multivariate models.

PECS hyper-demanding patients were nearly twice as likely to experience an in situ resolution (OR: 1.82; 95% CI: 1.24–2.67; χ^2^ = 9.41; *p* = 0.003). Similarly, when adjusted for provider type (ACT or ET), the results remained consistent, though markedly stronger for ET (OR: 5.11; 95% CI: 2.49–10.49; χ^2^ = 22.89; *p* < 0.001) compared to ACT (OR: 1.31; 95% CI: 0.70–2.44; χ^2^ = 0.72; *p* = 0.244).

### 3.2. Clinical Features

The group of PECS high-resource health system users had more comorbidities than the non-hyper-demanding group (OR: 2.12; 95% CI: 1.38–3.26; χ^2^ = 12.2; *p* < 0.001). The association between functional impairment and higher PECS demand was also evaluated. Overall, no significant association was found (OR: 1.18; 95% CI: 0.79–1.76; χ^2^ = 0.661; *p* = 0.416).

### 3.3. Andersen’s Model Components and Their Association with Health Care Utilization

A multivariate logistic regression model was constructed to assess which factors from Andersen’s behavioral model predicted in situ care resolution. The model included predisposing factors (age, sex), enabling factors (availability of a caregiver, type of resource), and clinical condition (comorbidity, dependency). PECS hyper-demand, male sex, and type of resource were significant predictors of in situ resolution, whereas clinical condition variables (comorbidity, dependency, and type of clinical process) were not significant and were excluded from the final model (Table 3).

Likewise, a multivariable linear regression was set up to determine which factors modulated the demand for PECS. Frequenting PHC services and the availability of a caregiver (facilitating factors) were introduced as predictors, together with the patient’s health needs, and age, sex, knowledge, and compliance with treatment (predisposing factors). Younger patients (predisposing factor), who had attended PHC more often (facilitating factor), with the presence of home oxygen therapy and more than three comorbidities (health needs), had a higher probability of requesting PECS (Table 4).

Lastly, the high-resource health system users’ profile was evaluated. The multivariate adjusted (age, multimorbidity, and smoking status) analysis showed that younger patients with more comorbidities, more visits to PHC in the previous six months, with home oxygen therapy, who were polymedicated and non-smokers had a higher risk of health care services consumption. There was no association between sex (predisposing factor), and the presence or absence of a family caregiver (facilitating factor), or living alone, poor functional status, or knowledge of the therapeutic regimen (clinical condition factors) (Table 5).

Finally, the factors that could be associated with frequenting PHC once receiving care from the PECS were explored. This model showed how patients who previously had a high frequency of PHC visits, who had a higher consumption of PECS, with more comorbidities, and availability of a family caregiver, were the ones who subsequently visited PHC services the most (Table 6).

## 4. Discussion

The aim of this study was to analyze the relationship between the number of people attending PHC in the population and chronic processes prior to the use of PECS and to verify whether the pattern of use of PHC services changes in the following six months. Considering Andersen`s model and its following adaptations [24,25,26], “clinical conditions and health needs” is the most determining factor for the activation of emergency resources among the older population with characteristics of comorbidity. The presence of cardiovascular and respiratory diseases, polypharmacy, and varying levels of comorbidity and functional decline are consistent with prior research [5]. Although polypharmacy was frequent, treatment adherence was good, contrasting with other studies [32]. In older adults, high comorbidity and reduced functional capacity often lead to increased use of emergency services, not necessarily because their needs are better met, but due to the limited availability of more appropriate alternatives to emergency care.

Predisposing factors included demographic characteristics such as age and sex. The median age exceeded 70 years, consistent with previous findings identifying age as a major determinant of health care demand [24,25,26]. However, unlike other studies where older age acted as a clear predisposing factor for emergency service use [33], our findings indicate that older patients were less likely to request out-of-hospital EMS. No significant sex-based differences were observed, although previous research has reported gender-related variations in both PHC and EMS utilization [34]. Regarding the finding that smokers made less use of PECS, it is very likely that patients with chronic conditions in more advanced stages (and therefore more frequent users) were former smokers, and that they quit smoking as their disease worsened.

Regarding enabling factors, older adults may have more difficulty activating EMS in acute situations, preferring traditional in-person care, resulting in a higher utilization of HEDs [35]. Social context and living conditions, such as living alone or being married, can also influence help-seeking behavior, in line with McCusker’s framework [26].

In view of the results obtained, this fact could justify the non-existence of significant changes in frequentation before and after contact with the PECS. PHC’s essential role in providing proactive care to prevent the need for HED care is based on the longitudinal and continuous care and support that GPs and PHCNs provide. It has been shown how these factors affect mortality, acute hospitalization, and the use of urgent care [36]. In our study, differences were even detected not only in care at the center, but also depending on whether they received nursing services at home.

The results indicate that the presence of a family caregiver is associated with increased utilization of PECS. This may be due to the fact that having someone dedicated to the patient’s care facilitates and encourages the pursuit of health care resources. Additionally, it is plausible that the presence of a family caregiver is more common in individuals with more severe health conditions or a higher level of dependency in activities of daily living, which may also be associated with greater illness-related disability.

According to our findings, the greater use of PECS in frequenters prior to PHC is associated with a prolongation of this intensive use of PHC afterwards. A greater use of PHC in the previous six months is not associated with a lower demand for PECS, as might be supposed a priori. It is possible that patients with multimorbidity have such a deteriorated clinical and functional situation that the impact of the care received at PHC is insufficient to cover their needs. However, the findings do not support this hypothesis, as those who most frequently used PECS were precisely the ones who did not require transfer to HEDs and whose care was resolved without acute hospitalization, despite no clinical or functional factors influencing the outcome. It appears that unmet needs in frequent PHC users may lead to increased PECS demand, without altering their PHC utilization patterns. This creates a vicious cycle that current research has yet to resolve [12,14,16].

The impact of interventions aimed at improving post-discharge coordination between HEDs and PHC is highly variable, with conflicting evidence regarding their effect on the reuse of emergency services, despite the wide range of strategies implemented [16,37]. Moreover, many of these interventions have been developed in contexts with limited PHC coverage or in health systems lacking universal access and public funding [9]. Notably, no comparable evidence exists on their impact on PECS use.

Finally, significant differences were found regarding the type of resource used (ET vs. ACT). A potential explanation for these findings may lie in the underlying cause of demand: patients attended by ETs were more likely to seek care for dyspnea, whereas ACT patients more frequently presented with cardiovascular conditions. As dyspnea is the most common reason for EMS use and is associated with critical diagnoses and poor prognosis [38], its presence may partially explain these differences, although they were not significant in the multivariable models. Further research is therefore needed to explore these findings.

In the case of this hyper-demanding population attended by EMS, most interventions are resolved in situ, reflecting the limited necessity for EMS activation. This may suggest that unmet needs in patients with an established pattern of frequent PHC use are redirected toward EMS demand. However, this does not subsequently alter their PHC utilization patterns, thereby generating a vicious cycle that current research has yet to determine how to address effectively [39].

Recent evaluations of the Spanish Chronic Care Strategy developed by the Ministry of Health [40] highlight that variability in the use of acute-care resources depends largely on institutional practices rather than on primary health care (PHC) activity levels. Nevertheless, strengthening PHC’s preventive and problem-solving role remains crucial, especially through home-based care, digital monitoring, and effective coordination between health and social care teams. Although access to diagnostic resources in PHC is widespread, the limited provision of home visits reveals an underused potential for early intervention that could reduce avoidable activation of prehospital emergency care services (PECS). Advances, such as shared electronic health records, teleconsultations, and telemonitoring, have improved continuity of care, but information integration remains fragmented across regions. Enhancing real-time communication between PHC and emergency services, supported by interoperable data systems and multidisciplinary coordination, could help interrupt the current cycle of recurrent emergency demand and promote a more anticipatory and coordinated response for patients with complex chronic conditions.

The results of this study highlight the need to reinforce coordination mechanisms between PECS and PHC. Emergency nurses are in a key position to identify frequent users and communicate with PHC teams, in order to facilitate continuity of care and reduce unnecessary hospital transfers. Furthermore, it would be interesting to implement protocols that enhance information exchange and optimize resource use across these levels of care.

### Limitations

This is the first study of these characteristics in Spain and no international experiences have been found with comparable care contexts, which compromises its generalization to PECS that have not developed models that incorporate ACT or in which PHC is poorly developed.

The possibility of underreporting some variables in the electronic medical record in this study is quite low, since variables that are part of the PECS quality assurance system have been taken and, therefore, the professionals maintain fairly stable recording patterns in these variables. Furthermore, this is a retrospective study, so that associations found should be corroborated in future prospective studies. On the other hand, some variables from Andersen’s model related to socioeconomic factors (educational level, income, etc.) could not be collected because they were not available in the medical record

Finally, the study was carried out before the COVID-19 pandemic, and it is likely that the ensuing health emergency modified healthcare-seeking behavior among patients with chronic conditions, including their use of PECS.

## 5. Conclusions

This study concludes that there are determinants of the behavior of demand for PECS in the population with chronic processes associated with their comorbidity, polypharmacy, presence of home medical devices, and their history of frequenting PHC. These findings have relevant implications for clinical practice within the PECS setting. Emergency nurses should actively participate in developing communication protocols between emergency and primary care services, perform comprehensive assessments of patients’ previous health care utilization, and provide education to both patients and caregivers on effective multimorbidity management. Furthermore, the establishment of early notification mechanisms is essential to ensure continuity of care and to reduce inappropriate use of emergency services. Strengthening collaboration between emergency and primary care professionals, especially through nurse-led coordination and the creation of protocols, may help to reduce inappropriate emergency service demand. These strategies should be prioritized by policymakers as well as health care managers to improve continuity of care in multimorbid patients.

Future research should adopt mixed-method approaches that integrate quantitative analyses of health care utilization with qualitative inquiry into the experiences and perceptions of both patients and health care professionals. Such designs would enable a deeper understanding of the mechanisms underlying frequent PECS use among individuals with multimorbidity and inform the development of evidence-based, contextually adapted interventions to strengthen coordination and continuity of care between primary and emergency care settings.

## Figures and Tables

**Table 1:** Characteristics of the sample.

	N = 532 Median (IQR) or n (%)
Age	73 (18)
Female Gender	278 (52.3)
≥5 Drugs	414 (77.8)
Functional status for activities of daily living:	
*Full autonomy*	376 (70.7)
*Partial help*	135 (25.4)
*Total help*	21 (3.9)
Technical and medical devices:	
*Mobility aids*	100 (18.8)
*Urinary incontinency devices*	92 (17.3)
*Home oxygen therapy*	37 (7.0)
*Hearing or vision aids*	23 (4.4)
Treatment adherence	391 (73.5)
Knowledge of their therapeutic regimen	424 (79.7)
Family caregiver available	162 (30.5)
Presence of risk lifestyles or loneliness	162 (30.5)
Transfer to HEDs	365 (68.6)
Main chronic comorbidities:	
*Diabetes*	109 (20.5)
*Arrythmia/Acute Coronary Syndrome*	163 (30.6)
*Stroke*	83 (15.6)
*COPD*	80 (15.1)
*Epilepsy*	42 (7.9)
*Other*	55 (10.3)
PECS modality:	
*Emergency Team (ET)*	327 (61.5)
*Advanced Coordination Team (ACT)*	205 (38.5)

COPD: Chronic obstructive pulmonary disease; HED: Hospital emergency department; PECS: Prehospital emergency care system.

**Table 2:** Health services utilization.

PHC Frequentation		PECS Hyper-Demand Median (IQR) (n = 168)	No PECS Hyper-Demand Median (IQR) (n = 364)	*p* *	Cohen’s d (95% CI)
6 months before demand of PECS	FP	7 (8)	4 (6)	<0.001	0.68 (0.49 to 0.87)
	PHCN	4 (7)	2 (6)	<0.001	0.28 (0.09 to 0.47)
	CM	0 (0)	0 (0)	0.001	0.30 (0.11 to 0.48)
	SW	0 (0)	0 (0)	0.008	0.27 (0.08 to 0.45)
	Total PHC visits	12 (13.5)	8 (11)	<0.001	0.51 (0.32 to 0.70)
6 months after demand of PECS	FP	6 (8)	4 (6)	<0.001	0.60 (0.41 to 0.78)
	PHCN	4 (7)	2 (5)	0.001	0.24 (0.06 to 0.43)
	CM	0 (1)	0 (0)	0.137	0.10 (0.08 to 0.29)
	SW	0 (0)	0 (0)	0.043	0.22 (0.03 to 0.40)
	Total PHC visits	12 (16)	9 (12)	<0.001	0.48 (0.29 to 0.66)

FP: Family physician; PHCN: Primary health care nurse; CM: Case manager; SW: Social worker; PECS: Prehospital emergency care system; PHC: Primary health care. * Mann–Whitney U and Wilcoxon signed-rank tests.

**Table 3:** Multivariable logistic regression model evaluating the odds of transfer to hospital EDs.

	B	*p*	OR (95% CI)
Type of PECS: ET	2.67	<0.001	14.49 (9.04 to 23.23)
PECS hyper-demand	−0.85	0.001	0.43 (0.26 to 0.69)
≥4 comorbidities	−0.45	0.106	0.64 (0.37 to 1.10)
Male gender	−0.62	0.006	0.54 (0.34 to 0.84)
Constant	0.13	0.530	1.12

PECS: Prehospital emergency care system; ET: Emergency team; Hosmer–Lemeshow: χ^2^ = 8.96 *p* = 0.256; R^2^ Nagelkerke: 0.4; classification accuracy: 77.6%.

**Table 4:** Multivariable linear regression model to evaluate the demand of PECS.

				95% CI	
	B	β	*p*	LCI	UCI
Constant	2.32	-	<0.001	1.83	2.81
Total visits to PHC in the previous 6 months	0.14	0.39	<0.001	0.11	0.16
Age	−0.06	−0.15	<0.001	−0.09	−0.03
Home oxygen therapy	4.04	0.18	<0.001	2.39	5.68
≥4 comorbidities	1.85	0.13	0.001	0.79	2.90

PECS: Prehospital emergency care system; PHC: Primary health care; LCI: Lower confidence interval; UCI: Upper confidence interval. R^2^: 0.24 Durbin–Watson: 1.67; Highest VIF: 1.04; Lowest tolerance: 0.95.

**Table 5:** Multivariable logistic regression to evaluate the profile of PECS hyper-demand in patients with multimorbidity.

	B	*p*	OR (95% CI)
≥4 comorbidities	0.63	0.010	1.87 (1.16 to 3.01)
Age	−0.04	<0.001	0.96 (0.94 to 0.97)
PHC visits 6 months before PECS demand	0.03	<0.001	1.04 (1.02 to 1.06)
Home oxygen therapy	1.28	0.002	3.61 (1.63 to 8.02)
Polymedication	0.94	0.009	2.57(1.26 to 5.21)
Smoking	−0.80	0.009	0.45 (0.25 to 0.82)
Constant	2.10	0.004	8.16

Hosmer–Lemeshow: χ^2^ = 3.793; *p* = 0.877; AUC: 0.73.

**Table 6:** Multivariable linear regression to evaluate PHC utilization after receiving PECS.

				95% CI	
	B	β	*p*	LCI	UCI
Constant	7.67	-	<0.001	4.53	10.81
Total PHC visits in the past 6 months	0.44	0.50	<0.001	0.37	0.50
Number of PECS episodes	0.48	0.19	<0.001	0.29	0.67
Comorbidities	0.91	0.08	0.029	0.09	1.74
Family caregiver available	3.95	0.13	<0.001	1.93	5.98
Knowledge of therapeutic regimen	1.79	0.06	0.060	−0.07	3.64

PECS: Prehospital emergency care system; PHC: Primary health care; LCI: Lower confidence interval; UCI: Upper confidence interval. R^2^: 0.43; Durbin–Watson: 2.0; Highest VIF: 1.26; Lowest tolerance: 0.79.

## Data Availability

The data presented in this study are available on request from the corresponding author. The data are not publicly available due to privacy and ethical restrictions.

## Public Involvement Statement

No public involvement in any aspect of this research.

## Guidelines and Standards Statement

This manuscript was drafted against the STrengthening the Reporting of OBservational studies in Epidemiology (STROBE) for observational studies. A complete list of reporting guidelines can be accessed via the equator network: https://www.equator-network.org/.

## Abbreviations

The following abbreviations are used in this manuscript:

HEDs	Hospital Emergency Departments
PECS	Prehospital Emergency Care Services
PHC	Primary Health Care
ET	Emergency Team
ACT	Advanced Coordination Teams
SD	Standard Deviation
FP	Family Physician
PHCN	Primary Health Care Nurse
CMN	Case Manager Nurse
SW	Social Worker
MECR	Mobile Electronic Clinical Record
VIF	Variance Inflation Factor
IQR	Interquartile Range
OR	Odds Ratio
CI	Confidence Interval

## References

[1] Bujold M., Pluye P., Légaré F., Participatory Review Team (2022). Decision-making and related outcomes of patients with complex care needs in primary care settings: A systematic literature review with a case-based qualitative synthesis. BMC Prim. Care.

[2] Alonso-Claudio G., Moralejo-Alonso L. (2020). Herramientas para garantizar la continuidad asistencial en el paciente pluripatológico crónico. Reccmi.

[3] Hu J., Wang Y., Li X. (2020). Continuity of Care in Chronic Diseases: A Concept Analysis by Literature Review. J. Korean Acad. Nurs..

[4] Edwards S.T., Greene L., Chaudhary C., Boothroyd D., Kinosian B., Zulman D.M. (2022). Outpatient Care Fragmentation and Acute Care Utilization in Veterans Affairs Home-Based Primary Care. JAMA Netw. Open.

[5] Ioakeim-Skoufa I., González-Rubio F., Aza-Pascual-Salcedo M., Laguna-Berna C., Poblador-Plou B., Vicente-Romero J., Coelho H., Santos-Mejías A., Prados-Torres A., Moreno-Juste A. (2024). Multimorbidity patterns and trajectories in young and middle-aged adults: A large-scale population-based cohort study. Front. Public Health.

[6] Bell C., Prior A., Frølich A., Appel C.W., Vedsted P. (2022). Trajectories in Outpatient Care for People with Multimorbidity: A Population-Based Register Study in Denmark. Clin. Epidemiol..

[7] Maun A., Björkelund C., Arvidsson E. (2023). Primary care utilisation, adherence to guideline-based pharmacotherapy and continuity of care in primary care patients with chronic diseases and multimorbidity—A cross-sectional study. BMC Prim. Care.

[8] Bellass S., Scharf T., Errington L., Bowden Davies K., Robinson S., Runacres A., Ventre J., Witham M.D., Sayer A.A., Cooper R. (2024). Experiences of hospital care for people with multiple long-term conditions: A scoping review of qualitative research. BMC Med..

[9] MacRae C., Fisken H.W., Lawrence E., Connor T., Pearce J., Marshall A., Lawson A., Dibben C., Mercer S.W., Guthrie B. (2022). Household and area determinants of emergency department attendance and hospitalisation in people with multimorbidity: A systematic review. BMJ Open.

[10] Rosell Ortiz F. (2020). Radiografía de los servicios prehospitalarios de emergencias. Emergencias.

[11] Consejería de Salud (2018). Junta de Andalucía Desarrollo de los Equipos Movilizables de Cuidados Avanzados (EMCA) en el SSPA. https://www.sspa.juntadeandalucia.es/servicioandaluzdesalud/sites/default/files/sincfiles/wsas-media-mediafile_sasdocumento/2019/desa_eqmovcuid.pdf.

[12] Wang C., Kuo H.-C., Cheng S.-F., Hung J.-L., Xiong J.-H., Tang P.-L. (2020). Continuity of care and multiple chronic conditions impact frequent use of outpatient services. Health Inform. J..

[13] Johnson R., Chang T., Moineddin R., Upshaw T., Crampton N., Wallace E., Pinto A.D. (2024). Using Primary Health Care Electronic Medical Records to Predict Hospitalizations, Emergency Department Visits, and Mortality: A Systematic Review. J. Am. Board. Fam. Med..

[14] Joseph S., Tomaschek R., Hug B.L., Beeler P.E. (2024). Enhancing communication and care coordination: A scoping review of encounter notification systems between emergency departments and primary care providers. Int. J. Med. Inform..

[15] Wackers E.M.E., Stadhouders N.W., Maessen M.F.H., Tanke M.A.C., Gaakeer M.I., van Dulmen S.A., Jeurissen P.P.T. (2023). Association between acute care collaborations and health care utilization as compared to stand-alone facilities in the Netherlands: A quasi-experimental study. Eur. J. Emerg. Med..

[16] Aghajafari F., Sayed S., Emami N., Lang E., Abraham J. (2020). Optimizing emergency department care transitions to outpatient settings: A systematic review and meta-analysis. Am. J. Emerg. Med..

[17] Memedovich A., Asante B., Khan M., Eze N., Holroyd B.R., Lang E., Kashuba S., Clement F. (2024). Strategies for improving ED-related outcomes of older adults who seek care in emergency departments: A systematic review. Int. J. Emerg. Med..

[18] Baier N., Geissler A., Bech M., Bernstein D., Cowling T.E., Jackson T., van Manen J., Rudkjøbing A., Quentin W. (2019). Emergency and urgent care systems in Australia, Denmark, England, France, Germany and the Netherlands—Analyzing organization, payment and reforms. Health Policy.

[19] Huntley A., Lasserson D., Wye L., Morris R., Checkland K., England H., Salisbury C., Purdy S. (2014). Which features of primary care affect unscheduled secondary care use? A systematic review. BMJ Open.

[20] Barrio Cortes J., Suárez Fernández C., Bandeira de Oliveira M., Beca Martínez M.T., Lozano Hernández C., del Cura González I., Barrio Cortes J., Suárez Fernández C., Bandeira de Oliveira M., Beca Martínez M.T. (2019). Utilización de los servicios de salud de Atención Primaria en los pacientes crónicos según nivel de riesgo. Revista Española de Salud Pública.

[21] Consejería de Salud y Consumo (2024). Plan Andaluz de Atención a la Cronicidad 2025–2028.

[22] Coca Boronat E., Díaz Pérez M.Á., Lupiáñez Pérez I., Pérez Ardanaz B., Fuentes Ruíz J.Á., Morales Asencio J.M. (2020). Prevalence of out-of-hospital nursing diagnosis for patients with chronic conditions: Improving our understanding of complexity. Emergencias.

[23] Montero García A., Morales Asencio J.M., Trujillo Illescas J.A., Martí C. (2016). Factors related to lack of autonomous mobility during out-of-hospital emergency care. Emergencias.

[24] Andersen R.M. (1995). Revisiting the behavioral model and access to medical care: Does it matter?. J. Health Soc. Behav..

[25] Gruneir A., Silver M.J., Rochon P.A. (2011). Emergency department use by older adults: A literature review on trends, appropriateness, and consequences of unmet health care needs. Med. Care Res. Rev..

[26] McCusker J., Karp I., Cardin S., Durand P., Morin J. (2003). Determinants of emergency department visits by older adults: A systematic review. Acad. Emerg. Med..

[27] Gutiérrez-Valencia M., Aldaz Herce P., Lacalle-Fabo E., Contreras Escámez B., Cedeno-Veloz B., Martínez-Velilla N. (2019). Prevalence of polypharmacy and associated factors in older adults in Spain: Data from the National Health Survey 2017. Med. Clin..

[28] González N., Bilbao A., Forjaz M.J., Ayala A., Orive M., Garcia-Gutierrez S., Hayas C.L., Quintana J.M., OFF (Older Falls Fracture)-IRYSS Group (2018). Psychometric characteristics of the Spanish version of the Barthel Index. Aging Clin. Exp. Res..

[29] Moorhead S., Swanson, Johnson M., Maas M. (2019). Clasificación de Resultados de Enfermería (NOC). Medición de Resultados en Salud.

[30] Brodeur M., Margo-Dermer E., Chouinard M.-C., Hudon C. (2020). Experience of being a frequent user of primary care and emergency department services: A qualitative systematic review and thematic synthesis. BMJ Open.

[31] Andalusian Health Service Plurypathologic Patient. https://www.sspa.juntadeandalucia.es/servicioandaluzdesalud/profesionales/cartera-de-servicios/atencion-primaria/i-area-de-atencion-la-persona/2-atencion-especifica/22-atencion-problemas-cronicos/227-paciente-pluripatologico.

[32] Walsh C.A., Cahir C., Bennett K.E. (2021). Longitudinal Medication Adherence in Older Adults with Multimorbidity and Association with Health Care Utilization: Results from the Irish Longitudinal Study on Ageing. Ann. Pharmacother..

[33] Haraldseide L.M., Sortland L.S., Hunskaar S., Morken T. (2020). Contact characteristics and factors associated with the degree of urgency among older people in emergency primary health care: A cross-sectional study. BMC Health Serv. Res..

[34] Aalam A.A., Iftikhar N., Baskaran N., Bhat A. (2024). Exploring Gender Disparities in Emergency Department Utilization: A Comprehensive Comparative Analysis of the Frequency of Female Versus Male Emergency Department Visits. Cureus.

[35] Sarbay İ., Mercan Baspinar M., Erman E., Cavdar B., Tamer I., Adiyaman A.M., Kosali K., Aksuz M.Y., Basat O., Calik M. (2025). Does Patient Satisfaction with Primary and Emergency Care Influence Non-Urgent Emergency Department Utilization? A Path Analysis. J. Multidiscip. Healthc..

[36] Sandvik H., Hetlevik Ø., Blinkenberg J., Hunskaar S. (2022). Continuity in general practice as predictor of mortality, acute hospitalisation, and use of out-of-hours care: A registry-based observational study in Norway. Br. J. Gen. Pract..

[37] Roberts T., Taylor C., Carlton E., Booker M., Voss S., Trevett N., Wattley D., Benger J. (2025). Emergency department interventions and their effect on subsequent healthcare resource use after discharge: An overview of systematic reviews. Scand. J. Trauma Resusc. Emerg. Med..

[38] Kauppi W., Herlitz J., Magnusson C., Palmér L., Axelsson C. (2020). Characteristics and outcomes of patients with dyspnoea as the main symptom, assessed by prehospital emergency nurses—A retrospective observational study. BMC Emerg. Med..

[39] Jimenez G., Matchar D., Koh G.C.-H., Car J. (2021). Multicomponent interventions for enhancing primary care: A systematic review. Br. J. Gen. Pract..

[40] Ministerio de Sanidad (2021). Estrategia para el Abordaje de la Cronicidad en el Sistema Nacional de Salud. Informe de evaluación y líneas prioritarias de actuación 2021.

